# Effects of Claudin-1 on the Action of *Clostridium perfringens* Enterotoxin in Caco-2 Cells

**DOI:** 10.3390/toxins11100582

**Published:** 2019-10-09

**Authors:** Iman Mehdizadeh Gohari, Jihong Li, Mauricio Navarro, Francisco Uzal, Bruce McClane

**Affiliations:** 1 Department of Microbiology and Molecular Genetics, University of Pittsburgh School of Medicine, Pittsburgh, PA 15219, USA; IMEHDIZA@pitt.edu (I.M.G.); jihongli@pitt.edu (J.L.); 2 California Animal Health and Food Safety Laboratory System, School of Veterinary Medicine, University of California Davis, San Bernadino, CA 92408, USA; mnavarrob@ucdavis.edu (M.N.); fauzal@UCDAVIS.EDU (F.U.)

**Keywords:** *Clostridium perfringens*, enterotoxin, claudins, occludin, tight junctions, diarrheal disease

## Abstract

*Clostridium perfringens* enterotoxin (CPE) contributes to diarrhea and an often-lethal enterotoxemia. CPE action starts when it binds to claudin receptors, forming a small complex (90 kDa). Six small complexes then oligomerize to create prepores, followed by insertion of beta-hairpins from CPE to form beta-barrel pores named CH-1 or CH-2. Of the ~27 members of the human claudin protein family, only some bind CPE. However, both receptor claudins and the nonreceptor claudin-1 (CLDN-1) are associated with the small and CH-1/CH-2 CPE complexes. Therefore, this study evaluated whether claudin-1 affects CPE action by generating a CLDN-1 null mutant in Caco-2 cells using CRISPR-Cas9. Compared to wild-type Caco-2 cells, paracellular permeability of the CLDN-1 mutant was significantly enhanced, suggesting that claudin-1 may reduce CPE absorption during enterotoxemia. The CLDN-1 mutant was also markedly more sensitive than wild-type Caco-2 cells to apically-applied CPE. The mechanism behind this increased sensitivity involved higher CPE binding by the CLDN-1 mutant vs. wild-type Caco-2 cells, which led to more CH-1/CH-2 complex formation. However, the CH-1/CH-2 complexes formed by the CLDN-1 mutant were less stable or trypsin resistant than those of wild-type cells. These results indicate that, although a nonreceptor, CLDN-1 positively and negatively influences CPE action.

## 1. Introduction

*Clostridium perfringens* type F strains, which by definition must produce *C. perfringens* enterotoxin (CPE), rank among the most common human enteric pathogens [1,2,3]. These Gram-positive anaerobes cause *C. perfringens* type F food poisoning, which is the second most common bacterial foodborne illness and affects about 1 million people per year in the USA [4]. Type F strains also cause up to 15% of all cases of nonfoodborne human gastrointestinal diseases, including antibiotic-associated diarrhea [5]. Type F infections can be fatal in the elderly or in people with preexisting fecal impaction/severe constipation [6,7]. Animal model studies strongly suggest that the absence of diarrhea in people with fecal impaction/severe constipation prolongs contact between the enterotoxin and their intestines, increasing CPE absorption into the circulation to cause an often-fatal enterotoxemia involving organs such as the liver [8].

CPE production is essential for the intestinal virulence of type F strains [9]. This enterotoxin is a 35 kDa protein that belongs to the aerolysin family of pore-forming toxins [10,11]. In sensitive host cells, such as human enterocyte-like Caco-2 cells, CPE action begins with its binding to receptors, which include a subset of the ~27-member human claudin protein family that plays an important role in maintaining the barrier and gating properties of mammalian tight junctions (TJs) [3,12,13].

Claudins possess two extracellular loops (ECLs), with both ECL-1 and ECL-2 participating in CPE binding [12,14]. While the ECL-1 sequence is largely conserved amongst all claudins, there is more variability among ECL-2 sequences. Consequently, only certain claudins possess ECL-2 sequences favorable for CPE binding. ECL-2 sequence variations also impact the CPE binding affinity among different receptor claudins. Consequently, some receptor claudins, like claudin-3 and -4, bind CPE strongly, while other claudins, like claudin-8 and -14, bind CPE less tightly [12]. Still other claudins, like claudin-1 (CLDN-1), are not CPE receptors because they lack a ECL-2 sequence favorable for CPE binding [12].

**Figure 1:**
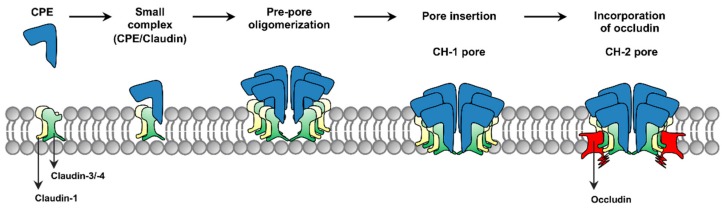
Formation of *Clostridium perfringens* enterotoxin (CPE) complexes. CPE (blue) binds to a receptor claudin (green, e.g., claudin-3 or -4) to form a small complex that also contains the nonreceptor claudin-1 (yellow). Six small complexes assemble into a prepore on the membrane surface. In the prepore, CPE extends beta-hairpins to form a beta-barrel pore complex named CH-1. Occludin (red) can also associate with CH-1 to form a second pore complex named CH-2. Based upon [15,16,17].

The current model for formation of CPE complexes is shown in Figure 1. Upon binding to a claudin receptor on host cells, CPE becomes sequestered in an ~90 kDa small complex that contains CPE, a claudin receptor and the nonreceptor CLDN-1 [15]. Approximately six small complex-associated CPE molecules then oligomerize to form a prepore on the host plasma membrane surface [15]. When each CPE monomer in this prepore extends a beta-hairpin [16], this results in formation of a beta-barrel pore named CH-1 [15]. A second large CPE pore complex named CH-2, which contains receptor claudins, CLDN-1 and the tight junction protein occludin, can also form in Caco-2 cells [15,17]. Thus, throughout its action, CPE remains closely associated with both claudin receptors and CLDN-1 [15].

Once formed, CPE pores become permeable to small molecules, particularly cations such as Ca^2+^ [3,18]. Treating host cells with low CPE concentrations creates only a small number of pores, causing a relatively limited Ca^2+^ influx that induces a modest calpain activation and caspase-3-mediated apoptosis [18,19]. Treatment with higher CPE concentrations leads to the formation of many pores, causing an even stronger Ca^2+^ influx and greater calpain activation that results in cell death from necrosis [18,19].

Animal model studies demonstrated that, in the small intestine, CPE causes intestinal damage that includes mucosal necrosis and villus blunting [20]. Those studies also strongly suggested this damage is important for CPE-induced diarrhea since, (i) the onset of CPE-induced intestinal damage coincides with the development of luminal fluid and electrolyte accumulation [21] and (ii) luminal fluid accumulation only occurs using CPE doses that cause this intestinal damage [22]. CPE-induced cytotoxicity appears to be important for the development of intestinal damage since a non-toxic CPE variant that binds to receptors but cannot form pores is also unable to cause significant intestinal damage or fluid accumulation in rabbit small intestinal loops [20]. Interestingly, CPE effects on paracellular permeability may contribute to enterotoxemia since, in a mouse model, measurable CPE levels are present in blood prior to the onset of intestinal damage [8].

The persistent association between CLDN-1 and the CPE complexes formed in Caco-2 cells is intriguing for several reasons. First, CLDN-1 has potent barrier-forming ability and thus is considered an important contributor to TJ integrity [23,24,25,26]. Therefore, interactions between CLDN-1 and CPE complexes might affect paracellular permeability or cytotoxicity in Caco-2 cells. Second, CLDN-1 is expressed in the intestines [23,24], so characterizing interactions between CLDN-1 and CPE is likely relevant for understanding CPE action in vivo. In response, the current study constructed in human enterocyte-like Caco-2 cells an isogenic CLDN-1 knockout mutant and complementing strain and used those cells to assess whether the presence of CLDN-1 affects the interactions of CPE with Caco-2 cells.

## 2. Results

### 2.1. Generation and Establishment of a CLDN-1 Knockout Mutant of Caco-2 Cells Using the CRISPR-Cas9 System

**Figure 2:**
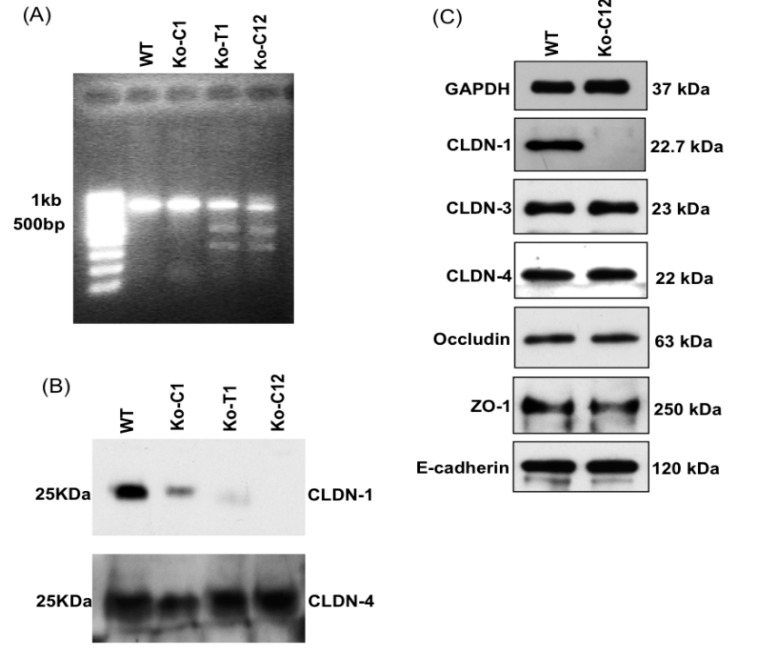
Establishment of claudin-1 (CLDN-1) knockout clones in Caco-2 cells. (**A**) Genomic DNA isolated from wild-type Caco-2 cells or CLDN-1 knockout clones was subjected to PCR for amplification of the CRISPR-Cas9 targeting site in the *cldn-1* gene. After purifying and annealing the PCR products, the products were incubated with T7 endonuclease I, which recognizes and cleaves mismatched DNA. Two shorter bands of the predicted size were generated in some clones, indicating that CRISPR-Cas9 had successfully introduced indel mutations at the targeted *cldn-1* site. This analysis indicated that the Ko-C1 clone contains only WT alleles, but the Ko-T1 and Ko-C12 clones had deletion alleles. (**B**) Immunoblots comparing CLDN-1 and CLDN-4 production by control Caco-2 cells and CLDN-1 knockout clones. These results indicated that the Ko-C12 clone completely lost the ability to produce CLDN-1, so this clone was selected for further experiments. (**C**) Immunoblot analysis comparing production of other tight junction proteins, including claudin-3 (CLDN-3), claudin-4 (CLDN-4), occludin and ZO-1, by CLDN-1 mutant cells vs. wild-type Caco-2 cells. GAPDH and E-cadherin were used as endogenous controls. For these blots, target proteins in cell lysates (30 µg protein/sample) were detected by Western blotting using specific primary antibodies and suitable horseradish peroxidase-labeled secondary antibodies. Results shown are representative of three repetitions.

To inactivate the *cldn-1* gene in Caco-2 cells, we used a plasmid carrying the all-in-one sgRNA targeted to the human *cldn-1* gene, which was purchased from GeneCopoeia. This construct was transfected into Caco-2 cells and transfectants were cultured for 2–3 weeks under selective pressure with 0.5 µg/mL neomycin. At that time, three independent clones (Ko-C1, Ko-T1 and Ko-C12) were obtained. The T7 endonuclease I assay, which only identifies and cuts mismatched DNA, was then performed on these clones to validate the presence of an indel mutation at the targeted site. The result of this experiment revealed that Cas9 had successfully introduced an indel mutation at the targeted *cldn-1* sites in two clones, named Ko-T1 and Ko-C12 (Figure 2A). Furthermore, immunoblot analysis indicated that the Ko-C12 clone has completely lost the ability to produce the CLDN-1 protein (Figure 2B), whereas it still produced other proteins, including several TJ proteins, at the same level as wild-type Caco-2 cells (Figure 2C). In addition, to verify the mutation of the Cas9 targeting site in the *cldn-1* gene of Ko-C12 clone, the PCR product from the *cldn-1* gene was also sequenced, which identified the expected deletion in the *cldn-1* gene (not shown). Therefore, this clone was selected for use in subsequent experiments.

### 2.2. Re-Expression of CLDN-1 after Complemention of the CLDN-1 Knockout Mutant

**Figure 3:**
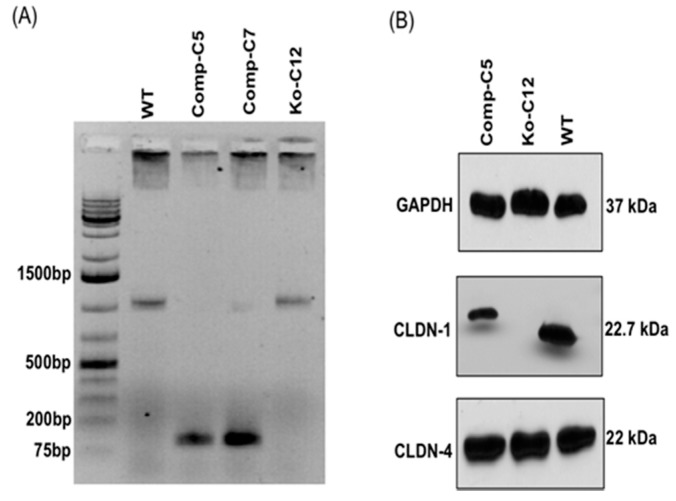
Establishment of CLDN-1 complementing clones in Caco-2 cells. (**A**) Confirmation of CLDN-1 complementing clones by PCR. For this experiment, the primers were designed based on *cldn-1* cDNA. Since the cDNA consists only of an exon, complementation of the *cldn-1* mutant results in genomic DNA that supports PCR amplification of a smaller band than wild-type or mutant genomic DNA, which also includes an intron. The first band on the left shows a 1 Kb DNA ladder (Thermo-Fisher). PCR analysis indicated that both the Comp-C5 and Comp-C7 clones were successfully complemented; the Comp-C5 clone was selected for further experiments. (**B**) Immunoblot analysis of CLDN-1 expression by the chosen Comp-C5 complementing cells. GAPDH and CLDN-4 were used as loading controls. Target proteins in cell lysates (30 µg protein/sample) were detected by Western blotting using specific primary antibodies and suitable horseradish peroxidase-labeled secondary antibodies. Results shown are representative of 3 repetitions.

To address potential concerns in later experiments whether secondary mutations might be responsible for any observed phenotypes of the CLDN-1 mutant, production of the CLDN-1 protein was restored to this mutant by complementation. The complemented CLDN-1 stable transfectants were established by transfection of the mutant with a plasmid encoding human *cldn-1* cDNA. After 2–3 weeks of culturing with 1.5 mg/mL of G418, multiple *cldn-1* complemented clones were obtained including Comp-C5 and Comp-C7. PCR analysis revealed that both clones (Comp-C5 and -C7) were successfully complemented (Figure 3A). In addition, Western blot analyses indicated that the stable transfectant Comp-C5 produced comparable amounts of CLDN-1 as did wild-type Caco-2 cells (Figure 3B). Because of a C-terminal Myc-DDK tag, the CLDN-1 protein made by the complemented cells has a slightly larger molecular weight than the CLDN-1 made by wild-type Caco-2 cells. As expected, the complemented CLDN-1 clone demonstrated claudin-4 (CLDN-4) and GAPDH expression by immunoblot analysis at levels similar to those in wild-type Caco-2 cells (Figure 3B).

### 2.3. Effects of the CLDN-1 Protein on Tight Junction Barrier and Paracellular Permeability Properties in Caco-2 Cells

**Figure 4:**
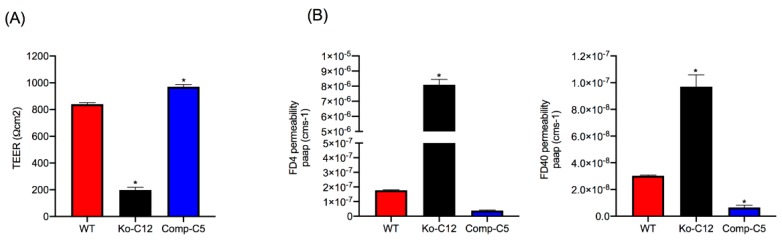
Effects of CLDN-1 on tight junction (TJ) electrical resistance and paracellular permeability properties of Caco-2 cells. (**A**) Effects of CLDN-1 on TJ electrical resistance. Wild-type Caco-2 cells, the CLDN-1 mutant or the CLDN-1-complemented Caco-2 cells (10^4^/1.12 cm^2^) were seeded on Transwell^®^ plates. After 14 days, Transepithelial Electrical Resistance (TEER) was measured in these confluent, polarized cultures. (**B**) Paracellular flux of FITC-labelled 4 kDa dextran (FD4, left) or FITC-labelled 40 kDa dextran (FD40, right). After 14 days of growth, Transwell^®^ cultures of confluent polarized wild-type Caco-2 cells, the isogenic CLDN-1 knockout mutant or CLDN-1 complemented Caco-2 cells were treated in the upper well chamber with HBSS containing FD4 or FD40 (final concentration of 1 mg/mL in HBSS buffer). After a 1 h incubation at 37 °C, the amount of FD4 or FD40 present in the basal chamber was measured using a BioTek Synergy fluorescence multi-plate reader. Data are presented as the means ± SD of three independent experiments. Asterisk represents *p* < 0.05 versus control.

CLDN-1 is considered an important structural and functional constituent of TJs in epithelial cells [23,24,27] and thus could impact the paracellular permeability of CPE during enterotoxemia, where CPE absorption begins prior to the onset of intestinal damage [8]. Therefore, the current study characterized the effects of CLDN-1 on barrier characteristics of Caco-2 cells. Initially this involved comparing the electrical resistance of barriers formed by polarized monolayers of wild-type Caco-2 cells versus similar monolayers of the isogenic derivative CLDN-1 mutant or CLDN-1 complemented cells when cultured for 2 weeks on Transwell^®^ filters. As shown in Figure 4A, cultures of the CLDN-1 mutant cells displayed a significant reduction in their Transepithelial Electrical Resistance (TEER) value compared to similar cultures of wild-type Caco-2 cells. Specifically, an average resistance of 197 ohms per cm^2^ was measured for the CLDN-1 mutant compared to 840 ohms per cm^2^ for wild-type Caco-2 cells. Interestingly, complementation of the CLDN-1 mutant to restore CLDN-1 production increased the resistance of the monolayer barrier above that of wild-type Caco-2 cells, with an average resistance of 970 ohms per cm^2^ (Figure 4A).

To assess the effects of CLDN-1 on macromolecule paracellular permeability, the next experiment compared the movement of 4 or 40 kDa fluorescein isothiocyanate (FITC)-labeled dextrans accrose confluent Transwell^®^ culture monolayers of wild-type Caco-2 cells or the isogenic derivatives. All cultures were all more permeable to the smaller 4 kDa FITC-labeled dextran (FD4) compared to the larger 40 kDa FITC-labeled dextran (FD40). However, consistent with their lower level of TEER, the polarized monolayers of the CLDN-1 knockout mutant showed a significantly increased transepithelial flux of both apically-applied FD4 and FD40 FITC-dextrans compared to wild-type Caco-2 cells (Figure 4B), indicating that the absence of CLDN-1 production increases the macromolecule paracellular permeability properties of Caco-2 cells. Wild-type Caco-2 cells exhibited more FD4 paracellular permeability than CLDN-1-complemented cells (Figure 4B), although this effect did not reach statistical significance. However, consistent with their higher TEER, the paracellular flux value of FD40 for polarized monolayers of CLDN-1-complemented cells was significantly reduced compared to similar cultures of wild-type Caco-2 cells (Figure 4B).

### 2.4. Comparison of CPE Cytotoxic Effects on Transwell^®^ Cultures of Wild-Type Caco-2 Cells vs. the Isogenic CLDN-1 Knockout Mutant and CLDN-1 Complemented Cells

**Figure 5:**
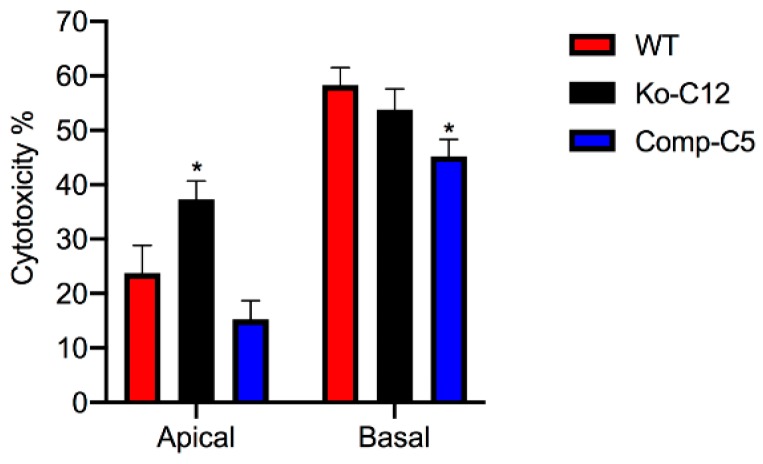
Comparison of cytotoxicity after apical vs. basolateral surface CPE treatment of confluent Transwells^®^ cultures of wild-type Caco-2 cells or the CLDN-1 mutant or CLDN-1 complemented cells. The specified cell types were seeded (10^4^ cells/well) into 12 mm^2^ Transwell^®^ plates. After 14 days, the confluent, polarized cultures were treated on their apical (upper well) or basolateral (bottom well) surface for 1 h at 37 °C with 1 µg/mL of purified native CPE. After completion of this treatment, the supernatants of either the apical or basal compartments of individual Transwell^®^ chambers for each specified cell type was collected. Using those supernatants, the cytotoxic effects of CPE was determined using a LDH cytotoxicity assay. Shown is the average of three independent experiments. Asterisk represents *p* < 0.05 versus control.

To evaluate whether the presence of CLDN-1 affects CPE cytotoxic activity, 1 µg/mL of purified CPE (a physiologic concentration of this toxin [28]) was added to polarized cultures of wild-type Caco-2 cells or isogenic derivatives grown in Transwell^®^ cultures and cytotoxic effects were then compared using an LDH-release assay. As shown in Figure 5, the isogenic CLDN-1 mutant exhibited significantly more LDH-release compared to wild-type Caco-2 cells or complemented cells when the 1 µg/mL CPE concentration was added to their apical surface, which mimics natural disease where CPE first interacts with the apical epithelial surface of the intestines.

It was reported previously [29] that wild-type Caco-2 cells are even more sensitive when CPE is applied to their basolateral surface due to the presence of more claudin receptors that permit greater CPE binding. This conclusion was confirmed in the current study, which showed that the CLDN-1 mutant is also more sensitive to CPE when treated on the basolateral vs. apical surface (Figure 5). However, there were no significant differences in cytotoxicity between wild-type Caco-2 cells or complemented cells vs. the CLDN-1 mutant when CPE was added to their basolateral surfaces (Figure 5).

Overall, the Figure 5 results demonstrated that the presence of CLDN-1 reduces the cytotoxic effects of CPE, but only when CPE is applied to the apical surface of polarized enterocyte-like Caco-2 cells, mimicking what occurs to enterocytes during CPE-mediated disease.

**Figure 6:**
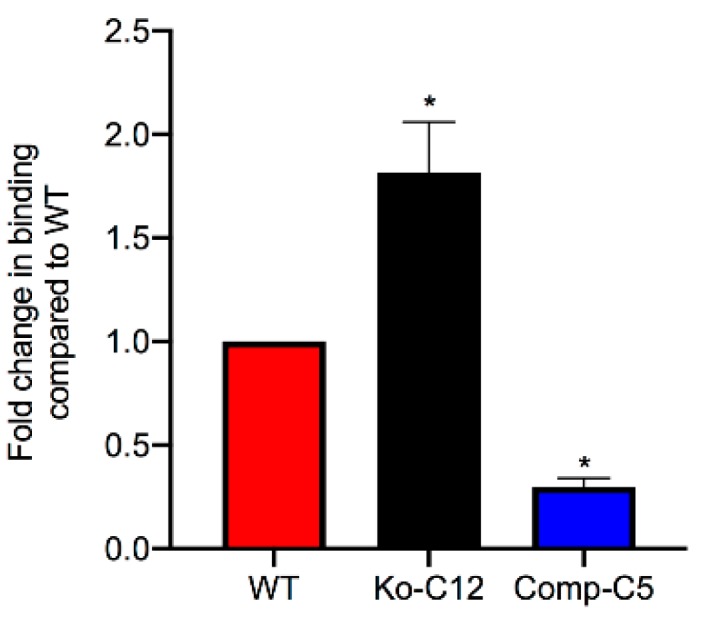
Effects of CLDN-1 on CPE binding to Caco-2 cells. To determine if the enhanced cytotoxicity observed in CLDN-1 knockout cells treated on their apical surface with CPE was due to an increase in CPE binding, each cell type (wild-type Caco-2 cells, the CLDN-1 mutant or the CLDN-1 complemented Caco-2 cells) was grown in 6-well Transwell^®^ plates. After two weeks, the confluent, polarized cells were treated on their apical surface for 1 h at 4 °C with 5 µg/mL of AF488-CPE. The cells were then rinsed three times with cold HBSS at 4 °C, collected, lysed, and fluorescence was measured using a BioTek Synergy fluorescence multi plate reader. Non-labeled CPE was used as a negative binding control. The experiment was performed in triplicate. Error bars indicate standard deviations of the means. Asterisk represents a significant (*p* < 0.05) difference compared to control Caco-2 cells treated with AF488-CPE.

The presence of CLDN-1 decreases the binding of CPE applied to the apical surface of polarized Caco-2 cells. To evaluate whether the cytotoxicity enhancement observed in Figure 5 when CPE is applied to the apical surface of CLDN-1 mutant cells involves increased CPE binding, polarized Transwell^®^ cultures of wild-type Caco-2 cells or the isogenic derivatives were treated on their apical surface at 4 °C with Alexa Fluor 488-labeled CPE (AF488-CPE), which shares similar cytotoxic activity as native CPE (data not shown). Results from this experiment (Figure 6) demonstrated that, compared to wild-type Caco-2 cells, the CLDN-1 mutant exhibited a significant, approximately two-fold increase in binding of apically-applied CPE. In contrast, complementation of the mutant caused a significant reduction in CPE binding even in comparison to wild-type Caco-2 cells (Figure 6). These findings provide evidence that the greater sensitivity of CLDN-1 mutant cells to apical CPE treatment involves an increase in CPE binding.

### 2.5. Comparison of CPE Large Complex Levels Formed in Wild-Type Caco-2 Cells vs. the Isogenic Derivatives

The results described above indicated that knockout of CLDN-1 production by Caco-2 cells led to more CPE binding and sensitivity when this toxin is apically applied, as occurs during gastrointestinal disease. Since CPE cytotoxicity for Caco-2 cells requires formation of CH-1 and CH-2 large pore complexes [30,31], an experiment compared formation of these two large CPE complexes after apical CPE treatment of Caco-2 cells vs. the isogenic derivatives.

**Figure 7:**
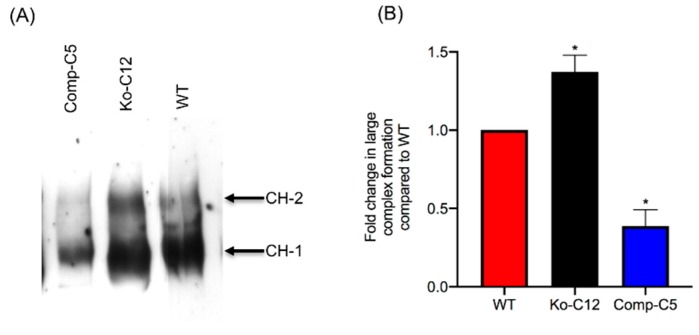
Comparison of CPE large complex levels formed in wild-type Caco-2, CLDN-1 mutant and complementing cells. (**A**) CPE Western blot analysis of CPE large complex levels. To assay for CPE large complex levels in each specified cell type, Transwell^®^ cultures of Caco-2 cells or the isogenic derivatives were treated on their apical surface with 1 µg/mL purified native CPE for 20 min at 37 °C. To detect CPE large complexes, cell lysates (10 µg of total protein/sample) were subjected to CPE Western blot analysis using rabbit polyclonal anti-CPE antiserum. (**B**) The levels of total large CPE complex formation in wild-type Caco-2 cells vs. the isogenic derivatives was determined by densitometry using Image J software. The experiment was performed at least three times. Error bars indicate standard deviations of the means. An asterisk indicates a statistically significant (*p* < 0.05) difference in large complex formation compared to control Caco-2 cells.

For this experiment, polarized Transwell^®^ cultures of the wild-type Caco-2 cells, the CLDN-1 mutant or the CLDN-1 complemented cells were apically treated with 1 µg of CPE/mL at 37 °C for 20 min. CPE Western immunoblotting of those cells then demonstrated that, under these treatment conditions, more of both CPE large complexes were present in CLDN-1 mutant cells than in wild-type Caco-2 cell (Figure 7A). Those differences in large complex formation levels reached statistical significant (Figure 7B). There was also a significant reduction in CPE large complex formation in CLDN-1 complemented cells compared to control Caco-2 cells (Figure 7A,B).

### 2.6. Comparison of CPE Large Complex Stability in Wild-Type Caco-2 Cells vs. the Isogenic Derivatives

**Figure 8:**
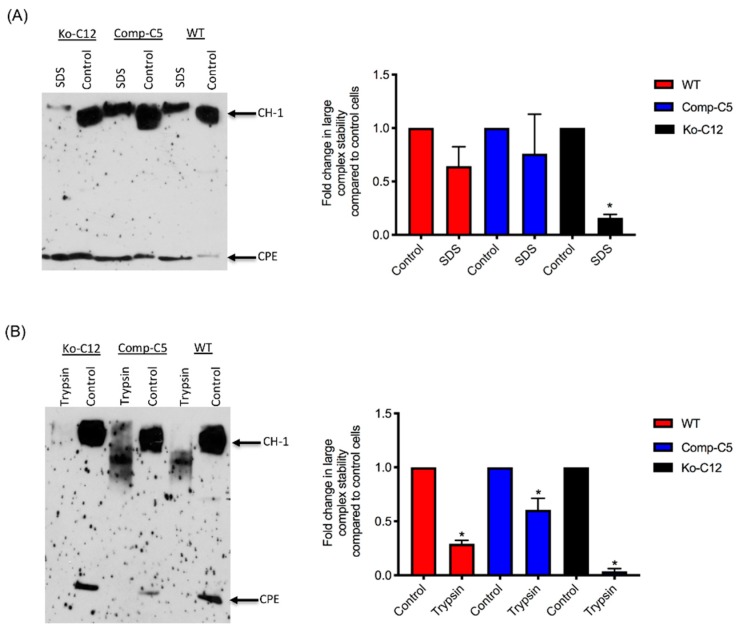
Comparison of the stability of the CPE large complexes made by wild-type Caco-2 cells, the CLDN-1 mutant and the complementing cells. (**A**, left panel) CPE Western immunoblot analysis of CPE large complex stability after SDS and heat treatment. To assay for stability of this large CPE complex in each specified cell type, Transwell^®^ cultures were treated with 1 µg/mL of purified native CPE for 1 h at 37 °C. To detect CPE large complexes, aliquots of cell lysates containing equalized amounts of CPE large complexes were subjected to CPE Western blot analysis using rabbit polyclonal anti-CPE antiserum. (**A**, right panel) Levels of CPE large complex in each specified cell type with or without SDS/heat treatment were compared by densitometry using Image J software. (**B**, left panel) CPE Western immunoblot analysis of CPE large complex stability after trypsin treatment. For this experiment, the large CPE complex for each specified cell type was prepared as described above and aliquots of cell lysates containing equalized amounts of CPE large complexes were or were not mixed with 30 µg/mL of trypsin and incubated for 30 min at 37 °C. The samples, treated or non-treated, were then heated to inactivate trypsin (if present) and CPE large complexes were detected by Western blot analysis using rabbit polyclonal anti-CPE antiserum. (**B**, right panel). The levels of CPE large complex remaining relative to the control (no trypsin) amount was determined by densitometry using Image J software. Both experiments were performed at least three times. Error bars indicate standard deviations of the means. An asterisk indicates a statistically significant (*p* < 0.05) difference in large complex stability compared to intact cells.

CPE large complexes exhibit exceptional resistance to heating in the presence of SDS and partial resistance to proteases [32]. Therefore, experiments evaluated if the presence of CLDN-1 affects CPE large complex stability. In these experiments, polarized Transwellc cultures of wild-type Caco-2 cells or the isogenic derivatives were treated on their apical surface with CPE (1 µg/mL) at 37 °C for 1 h, followed by treatment to cell extracts with SDS and heat treatment or with trypsin and heat treatment (to inactivate trypsin). The stability of CPE large complexes was then assessed by CPE Western blotting using 8% acrylamide gels to improve visualization of large complex breakdown or degradation. As shown in Figure 8A,B, the large CPE complexes formed by the CLDN-1 mutant were less stable to SDS and heat or trypsin and heat compared to the large complex of wild-type cells, indicating that CLDN-1 contributes to CPE large complex stability.

## 3. Discussion

CPE is an unusual toxin since both receptor and nonreceptor proteins are associated with its large (pore) complexes [15]. Those host proteins include receptor claudins, whose importance for CPE action is well established [12]. For example, cells such as fibroblasts that do not naturally produce any claudins are also unable to bind or respond to CPE [33,34]. However, when fibroblasts are transfected to produce various claudins, their CPE sensitivity correlates with the CPE binding affinity of the claudin expressed by that transfectant [34].

Nonreceptor host proteins are also associated with CPE complexes. For example, the TJ protein occludin is present in the CH-2 CPE pore complex made by Caco-2 cells [17]. Even more persistent is the association of the nonreceptor CLDN-1 with CPE complexes, i.e., CLDN-1 is associated with the small CPE complex, as well as both the CH-1 and CH-2 large CPE pore complexes [15]. Since CPE does not bind directly to occludin or CLDN-1 [17,34], the association of those tight junction proteins with CPE complexes likely involve their ability to interact with claudin receptors [35].

Despite those persistent associations between CLDN-1 and CPE, the functional consequences of such interactions had not been evaluated prior to the current study even though CLDN-1 is expressed in the intestines [23,24] and has excellent barrier-forming properties that contribute to the TJ seal [23,24,26,27,36]. Those CLDN-1 characteristics suggested that the association of this protein with CPE might impact CPE action, which was tested in the current study by comparing various effects of CPE on wild-type Caco-2 cells vs. isogenic CLDN-1 mutant or stable complementing cells. Results of these analyses indicated that CLDN-1 influences CPE action in several ways, some of which clearly enhance CPE cytotoxicity, and perhaps in vivo activity, while others may impede CPE toxicity during disease.

Inactivating the *cldn-1* gene in several mammalian cell lines has been reported to decrease their TJ seal [23,24,37]. However, to our knowledge, it has not yet been determined whether this relationship holds true for Caco-2 cells. Some data even question this relationship, since overexpression of CLDN-1 in Caco-2 cells did not affect paracellular permeability properties [38]. Consequently, the current work first evaluated whether CLDN-1 is a contributor to the sealing properties of the TJ in Caco-2 cells. TEER, a measure of TJ tightness and integrity [39], was reduced >4-fold when CLDN-1 production was eliminated in polarized Caco-2 cell cultures. This phenotype was specifically attributable to loss of CLDN-1 production since it was reversible by complementation. Consistent with CLDN-1 contributing to the TJ seal, polarized wild-type Caco-2 cells exhibited limited permeability to apically-applied FD4 dextran or (especially) FD40 dextran, but the CLDN-1 knockout mutant was >10 fold more permeable to these markers. This increased apical permeability to FITC-labeled dextrans was also specifically attributable to the absence of CLDN-1 since it was reversible by complementation of the CLDN-1 mutant. Notably, the complemented strain exhibited even higher TEER and permeability properties than wild-type Caco-2 cells, which may be explained by the presence of an epitope tag on the C-terminus of CLDN-1 made by the complemented cells. Since the C-terminal tail of claudins has a PDZ domain that mediates interaction with other proteins [24,36], e.g., ZO-1, it is possible that the presence of this epitope tag affects those interactions and impacts the tightness of the TJ seal.

This study did not attempt to compare the paracellular passage of CPE across polarized monolayers of wild-type Caco-2 cells vs. the isogenic derivatives because such experiments would be complicated by the cytotoxic effects of CPE, which would disrupt intact monolayers. However, the increased FD40 paracellular permeability properties of polarized CLDN-1 mutant monolayers suggests pathogenic relevance. For example, since the FD40 marker is approximately the same molecular mass as 35 kDa CPE, Figure 4 results suggest that, in the intestines, CLDN-1 could partially inhibit the development of enterotoxemia by impeding CPE entry into the bloodstream via the paracellular route. Paracellular passage of CPE appears to be important for enterotoxemia since i) CPE introduced into mouse small intestinal loops becomes detectable in the bloodstream of those mice even prior to the onset of gross intestinal damage [8] and ii) CPE is not internalized well, if at all, by host cells [40]. However, as discussed below, CLDN-1 also reduces CPE cytotoxicity so it is possible that, during enterotoxemia, this claudin may also inhibit any CPE uptake resulting from intestinal damage.

More commonly than enterotoxemia, type F strains cause CPE-induced diarrheal disease. As mentioned in Section 1, CPE-induced cytotoxicity apparently contributes to the intestinal histologic damage associated with the diarrheic symptoms of type F infections. Therefore, determining that CLDN-1 inhibits CPE-induced cytotoxicity for polarized, human enterocyte-like Caco-2 cells suggests possible pathophysiologic relevance. Supporting the possible relevance of CLDN-1 effects during disease, it is notable that this CLDN-1 effect only occurs when CPE is applied to the apical surface of polarized Caco-2 cells, which mimics what happens to intestinal epithelial cells during disease where CPE is released into the intestinal lumen by noninvasive type F sporulating cells and then interacts with the apical surface of the intestinal epithelium. To our knowledge, this is the first example of a host protein inhibiting the cytotoxic action of a bacterial pore-forming toxin.

This study also identified one mechanism to explain the reduction in CPE cytotoxicity caused by CLDN-1, i.e., the presence of CLDN-1 reduced the binding of apically-applied CPE to Caco-2 cells. This reduced CPE binding may be explained, at least in part, by the Figure 4 results showing that CLDN-1 impairs the paracellular transit of FD40 dextran, which is of similar molecular mass as CPE. Thus, when present, CLDN-1 should inhibit CPE access to the abundant claudin receptors present on the basolateral surface of Caco-2 cells [29]. Consequently, due to this reduced CPE binding, less CPE large pore complexes were detected in cells producing CLDN-1, so those cells showed less death. Consistent with this model is the increase in CH-2 formation observed for the CLDN-1 mutant vs. wild-type Caco-2 cells, which is notable since CH-2 contains occludin, which interacts much better with CPE when the toxin is applied basolaterally vs. apically [29]. Furthermore, almost no CH-2 complex was made in the complemented cells that form even tighter barriers than wild-type Caco-2 cells. Those CH-2 observations also indicate that the presence of CLDN-1 affects CPE interactions with other tight junction proteins besides receptor claudins. Last, no apparent differences were observed in migration of the CH-1 or CH-2 CPE complexes made by the CLDN-1 mutant, vs. parent Caco-2 cells on Western blots which is likely attributable to two factors. First, these complexes are very large, i.e., >~500 kDa or >600 kDa, respectively [15], so the absence of 23 kDa CLDN-1 may have minimal impact on the size of the CH-1 or CH-2 complexes (note that it is unknown how many copies of CLDN-1 are present in these complexes). Second, the CH-1 and CH-2 complexes migrate anomalously on SDS-PAGE [15], which may mask size contributions of CLDN-1 to these complexes.

All of the effects described above involve CLDN-1 inhibiting CPE cytotoxicity or paracellular permeability. Paradoxically, the presence of CLDN-1 was found to increase the stability of CPE large complexes formed in Caco-2 cells. For example, the CPE complexes made by wild-type or complemented Caco-2 cells were more resistant to heat and SDS than the complexes made by the CLDN-1 mutant. Of notable potential pathophysiologic relevance, CPE complexes formed in cells producing CLDN-1 also exhibited greater trypsin resistance. Since CPE complexes are formed in the intestines [8], where intestinal proteases are present, it is possible that CLDN-1-mediated protease resistance helps to protect the CPE large complexes, and thus enhance CPE activity, during intestinal disease.

Unfortunately, it is not straightforward to test directly whether the contradictory effects of CLDN-1 on CPE action in Caco-2 cells impact CPE toxicity during enterotoxemia or diarrheal disease. CLDN-1 knockout mouse are nonviable, dying within the first day of birth [27,41]. Therefore, more subtle in vivo studies will be needed in the future to tease out the in vivo relationship between CPE and CLDN-1.

## 4. Materials and Methods

### 4.1. Chemicals, Antibodies and Kits

Rabbit polyclonal antibodies 51-9000, 34-1700, 36-4800 against claudin-1, -3, -4, respectively, were purchased from ThermoFisher (Scientific Waltham, MA, USA). These antibodies were raised against synthetic peptides corresponding to unique epitopes present in the cytoplasmic C-terminal tail of each claudin. Purified mouse anti-E-cadherin (610182, BD Transduction Laboratories, San Jose, CA, USA), Rabbit anti-ZO-1 (61-7300, Zymed, San Francisco, CA, USA), Rabbit anti-GAPDH D16H11 (5174S, Cell Signaling, Danvers MA), and Rabbit anti-Occludin (71-1500, Zymed) were also used in this study. Rabbit polyclonal CPE antiserum had been prepared previously, as described [42]. Goat polyclonal anti-rabbit or anti-mouse horseradish peroxidase conjugate were purchased from Sigma (St. Louis, MO, USA). A T7 endonuclease I assay kit was purchased from GeneCopoeia (Rockville, MD, USA).

### 4.2. Cell Culture

Authenticated Caco-2 human colonic epithelial cells were routinely cultured in Eagle’s Minimum Essential Medium (Lonza. Walkersville, MD, USA) supplemented with 1% glutamine (Corning, Corning, NY, USA), 1% MEM nonessential amino acids (HyClone, Logan, UT, USA), 10% heat inactivated fetal bovine serum (Alphabioregen, Boston, MA, USA) and 100 µg/mL of penicillin-streptomycin (Corning). Caco-2 cells were grown at 37 °C in an atmosphere with 5% CO_2_. The CLDN-1 mutant and complementing cell lines, created as described later, were also cultured under the same culture conditions, except that the medium for CLDN-1 knockout mutant culture was supplemented with 0.5 µg/mL neomycin (Fisher Scientific, Waltham, MA, USA) and the medium for the complementing cell culture was supplemented with 1.5 mg/mL G418 (Fisher Scientific).

### 4.3. Enterotoxin

CPE was purified to homogeneity from type F strain ATCC^®^ 12916 (purchased from ATCC, Manassas, VA, USA), as described previously [43]. Purified CPE was fluorescently labeled using an Alexa Fluor^TM^ 488 (AF488) Protein Labeling Kit (ThermoFisher Scientific) as described previously [44]. When assayed with an LDH cytotoxicity detection kit (Roche, Basel, Switzerland), this AF488-labeled CPE retained similar cytotoxic properties as native CPE for Caco-2 cells (data not shown).

### 4.4. Plasmids

The CRISPR plasmid pCRISPR-CG01, an all-in-one sgRNA plasmid for inactivating the human *cldn-1* gene, was purchased from GeneCopoeia. The *cldn-1* gene target site is CACGATGTTGTCGCCGGCAT. The *cldn-1* complementing plasmid RC204466, which contains *cldn-1* cDNA with a C-terminal Myc-DDK tag, was purchased from Origene (Rockville, MD, USA).

### 4.5. Preparation of Stable Caco-2 CLDN-1 Knockout Transfectants and Complementing Cells

On day one, a culture of wild-type Caco-2 cells or the CLDN-1 knockout mutant was heavily split 1 to 1.5 in 6-well plate and then grown overnight to 70–90% confluence. A 5 µg aliquot of plasmid DNA (pCRISPR-CG01, for constructing the CLDN-1 mutant, or pRC204466, for constructing the complementing cells) was mixed with 5 µL of P3000 reagent (from the Lipofectamine 3000 transfection kit, ThermoFisher Scientific) and 110 µL of Opti-MEM medium (Gibco, Gaithersburg, MD, USA). In a separate tube, 7.5 µL of L3000 reagent (from the Lipofectamine 3000 transfection kit) was mixed with 110 µL of Opti-MEM medium. The two tubes were then mixed together and further incubated for 15 min at room temperature before the mix was added to a wild-type Caco-2 cell culture (when the mix contained pCRISPR-CG01) or a CLDN-1 knockout mutant cell culture (when the mix contained pRC204466) in a total volume of 1 mL of MEM. The cultures were incubated for 6 h at 37 °C and then fed with fresh cell culture medium. After 24 h, the cultures were trypsinized, plated at 1:10 dilutions, and grown under selective pressure with 0.5 µg/mL neomycin (to select for the claudin-1 mutant) or with 1.5 mg/mL G418 (to select for the complementing cell line). The cells were then grown, with selective pressure, for two to three weeks with a change of medium every 4 days. When colonies were discernible by the naked eye, they were transferred to a 48-well plate and culture was continued until enough cells were available to perform the T7 endonuclease I assay and CLDN-1 Western blot detection.

### 4.6. PCR Amplification of Genomic DNA

Genomic DNA of wild-type Caco-2 cell or the isogenic derivative CLDN-1 knockout mutant or CLDN-1 complemented cells were isolated using the DNeasy blood and tissue kit (Qiagen, Hilden, Germany) according to the manufacturer’s instructions. The isolated DNA was subjected to PCR using the primer set Forward: 5′-CTGGGAGCAACCGCAGCTTCTA-3′ and Reverse: 5′-CCCCAAATCTCGGAATGCCT-3′ to amplify a 789 bp product from *cldn-1* gene sequences for the T7 assay described below. To screen for complemented cells, PCR was performed using primers Forward: 5′-GCATGAAGTGTATGAAGTGCTTGGA-3′ and Reverse: 5′-CGATTCTATTGCCATACCATGCTG-3, which amplify a 132 bp product using *cldn-1* cDNA but, due to the presence of an intron, a 1087 bp product using DNA containing the wild-type *cldn-1* gene.

### 4.7. T7 Endonucleases I (T7EI) Assay

T7EI assay is commonly used to verify the mutations (deletion or insertion) obtained using CRISPR-Cas9 editing system [45]. This enzyme is able to accurately detect and cut the mismatched DNA. This assay was used to analyze mutation of the *cldn-1* gene in Caco-2 cells according to the manufacturer’s instructions (GeneCopoeia). Briefly, genomic DNA of CLDN-1 mutant cells was extracted and used as a PCR template for amplification of the *cldn-1* gene using specific primers mentioned above. Subsequently, the 789 bp PCR product was purified, denatured and re-hybridized to generate heteroduplex mismatches. The re-annealed PCR fragments were then incubated with T7EI which recognized and cleaved the mismatched DNA and generated two digested fragments (347 bp and 462 bp).

### 4.8. Western Blot Analysis of Caco-2 Cell Proteins

Wild-type Caco-2 cells or the isogenic derivatives were seeded into 100-mm^2^ diameter polycarbonate membrane Transwell^®^ plates (Corning Costa, Corning, NY, USA) and cultured for 14 days. Confluent polarized cultures were then washed once with Hanks’ balanced salt solution containing Ca^2+^ and Mg^2+^ (HBSS, Corning Cell Gro, Corning, NY, USA), scraped gently by a rubber policeman, and lysed with 300 µL of lysis buffer A (50 mM Tris-HCl, pH 7.4, 1% NP-40, 0.25% Na-deoxycholate, 150 mM NaCl, 1 mM EDTA, 1 mM Phenylmethylsulfonyl fluoride (PMSF) with protease inhibitor cocktail III for mammalian cells (RPI, Corp., Mount Prospect, IL, USA). The lysed cells were centrifuged and the supernatant collected. Total protein concentration in each supernatant was then quantified using the BCA protein assay kit (ThermoFisher Scientific) and 30 µg of supernatant protein from each cell lysate was loaded on a 10% acrylamide gel containing SDS. The Western blot was carried out as previously described [15] using primary and secondary antibodies described above.

### 4.9. Cytotoxicity

Wild-type Caco-2 cells, the CLDN-1 knockout mutant or the CLDN-1 complemented cells were seeded at a density of 10^4^ cells/well into 12-mm^2^ diameter polycarbonate membrane Transwell^®^ plates. The cells were grown for 14 days with a change of medium every 2 days. The cultures were then washed twice with warm HBSS buffer. Subsequently, 0.5 mL of warm HBSS containing 1 µg/mL of purified native CPE were added to the top or bottom chamber of each Transwell^®^ plate and incubated at 37 °C for 1 h. HBSS with or without 1% Triton X-100 (Sigma) were used as negative and positive controls, respectively. After this treatment, the supernatant was collected and cytotoxicity was assessed using an LDH cytotoxicity detection kit (Roche) according to the manufacturer’s instructions.

### 4.10. Trans-Epithelial Barrier Measurements (TEER) and Paracellular Permeability

Wild-type Caco-2 cells, or the isogenic derivative CLDN-1 mutant or CLDN-1 complemented cells, were seeded at a density of 10^4^ cells/well in 12-well (1.12 cm^2^, 0.4 µm pores) Transwell^®^ plates (Corning Costar) and grown as described above for two weeks. TEER was measured using Millicell^®^ ERS-2 (Electrical Resistance System) (Sigma) and calculated via the following equation.

Unit Area Resistance = Resistance (Ω) × Effective Membrane Area (cm^2^).

To measure paracellular permeability [46], the medium was gently removed and cells were washed twice with HBSS buffer added to both the upper and lower chambers. The inserts were transferred to fresh 12-well plates and 0.5 mL of HBSS containing either FD4 (Sigma Chemical, final concentration of 1 mg/mL) or FD40 (Sigma Chemical, final concentration of 1 mg/mL) were added into the upper chambers of Transwell^®^ plate. In addition, 1.5 mL of HBSS were added to the lower chamber of each well. After a 1 h incubation at 37 °C, the transferred FITC-dextran concentration in the lower chamber was determined using a BioTek Synergy fluorescence multi plate reader (Bio Tek, Winooski, VT, USA) with excitation and emission of 485 nm and 530 nm, respectively.

### 4.11. CPE Binding Assay

Wild-type Caco-2, CLDN-1 mutant, or CLDN-1 complemented Caco-2 cells were seeded at a density of 10^4^ cells/well in 6-well Transwell^®^ plates (Corning Costar) and grown for two weeks. The confluent polarized cultures were then washed twice with cold HBSS. Following the last wash, cells were treated for 1 h at 4 °C on their apical surface with 1.0 mL of cold HBSS containing 5.0 µg/mL of AF488-CPE. As a negative control, similar cultures were treated with 5.0 µg/mL of unlabeled CPE. After this treatment, the cells were rinsed three times with cold HBSS. The attached cells were then gently scraped and harvested by centrifugation. Subsequently, the cells were lysed with 200 µL of lysis buffer A with 2% Benzonase (Novagen, Madison, WI, USA) at 4 °C for 10 min. The lysed cells were centrifuged and supernatant retained. A 100 µL aliquot of supernatant was read using a BioTek Synergy fluorescence multi plate reader with excitation and emission of 485 nm and 530 nm, respectively. Background fluorescence (negative control) was subtracted from that of matching test sample.

### 4.12. Comparison of CPE Large Complex Formation Levels

Wild-type Caco-2 cells or the isogenic derivatives were grown in 100-mm^2^ Transwell^®^ plates to confluency (14 days old). The confluent polarized cultures were then rinsed twice with warm HBSS and treated on their apical surface with a 5.0 mL aliquot of HBSS containing 1 µg/mL of purified native CPE for 20 min at 37 °C. After this treatment, the cells were rinsed twice with HBSS to remove unbound toxin. Subsequently, the cells were gently scraped by a rubber policeman, collected by centrifugation and those cells were added to the detached cells collected during washing. The harvested cells were suspended and lysed in 1.0 mL of lysis buffer A (described above) for 30 min at 4 °C with gentle shaking. After this step, the lysed cells were centrifuged. Supernatants (10 µg protein) were then electrophoresed on a 6% SDS-PAGE gel, followed by electrotransfer onto a nitrocellulose membrane (Bio-Rad, Hercules, CA, USA) for CPE large complex Western blotting. Briefly, the membrane was blocked with 5% *w/v* nonfat dry milk dissolved in TBST buffer (Tris buffered saline, 0.1% Tween 20) for 1 h at room temperature. The blocked membrane was then probed with rabbit anti-CPE, 1:1000 in TBST buffer with 5% *w*/*v* nonfat dry milk, overnight at 4 °C. After three washes, the blots were incubated with horseradish peroxidase-conjugated goat anti-rabbit IgG secondary antibody (1:10,000 in TBST buffer with 5% *w/v* nonfat dry milk) for 1 h at room temperature. Following those washes, CPE large complexes were detected by SuperSignal West Pico substrate (ThermoFisher). CPE large complex levels in each specified cell type were compared by densitometry using Image J software (version 1.51J8, National Institutes of Health, Bethesda, MD, USA). The above procedure was repeated at least three times for each sample.

### 4.13. Analysis of CPE Large Complex Stability

To form CPE large complexes, Transwell^®^ cultures of each specified cell type were apically-treated with CPE as described above, with slight modification. For this experiment, the cells were treated with 5.0 mL of HBSS containing 1 µg/mL of purified CPE for 60 min at 37 °C. In addition, the cells were lysed with lysis buffer without EDTA, PMSF or protease inhibitors. Half of each sample was left intact and incubated at room temperature for 30–40 min, while the other half was used to assess CPE large complex stability. Two different approaches were used to evaluate this stability: (1) heating the samples, the presence of 10% SDS, at 100 °C in a heating block for 30 min or (2) incubating the samples with 30 µg/mL of trypsin from porcine pancreas (Sigma) for 30 min at 37 °C, followed by 10 min heating at 100 °C in a heating block to inactivate the enzyme. Those samples were then analyzed by CPE Western blotting, as described above, with slight modification. For this experiment, the samples, treated or non-treated, were electrophoresed side-by-side on an 8% acrylamide gel containing SDS to optimize visualization of degraded CPE large complexes. The amount of CPE large complex remaining relative to the starting amount was determined by densitometry using Image J software. The above procedure was repeated at least three times for each sample.

### 4.14. Statistical Analyses

All experiments presented in this study were repeated three times independently and values are shown as mean ± standard deviation (SD). For statistical analysis, one-way analysis of variance (ANOVA) with Tukey’s post hoc test was performed using GraphPad Prism, version 6 (GraphPad, San Diego, CA, USA).
